# Korean fermented soybean paste (*Doenjang*) has anti-obesity and anti-hypertensive effects via the renin-angiotensin system (RAS) in high-fat diet-induced obese rats

**DOI:** 10.1371/journal.pone.0291762

**Published:** 2023-10-20

**Authors:** Hayoung Woo, Anna Han, Jung Eun Park, Youn-Soo Cha

**Affiliations:** 1 Department of Food Science and Human Nutrition, Jeonbuk National University, Jeonju-si, Jeollabuk-do, Republic of Korea; 2 Department of Nutritional Sciences, University of Connecticut, Storrs, CT, United States of America; 3 K-Food Research Center, Jeonbuk National University, Jeonju-si, Jeollabuk-do, Republic of Korea; 4 Nutracore Co., Ltd., Suwon, Republic of Korea; Ehime University Graduate School of Medicine, JAPAN

## Abstract

The health-beneficial outcomes of *doenjang*, a Korean fermented food have been questioned due to its high salt content; moreover, the detailed underlying mechanisms of its health beneficial effects are not fully investigated. Thus, this study aimed to investigate *doenjang*’s anti-obesity effects, anti-hypertensive effects, and its underlying mechanisms in high-fat diet -induced obesity. Sprague-Dawley rats fed with normal diet (ND), high-fat diet (HD), HD with 8% additive salt (HDS), or HD with *doenjang* containing 8% salt (HDJ) for 13 weeks. Compared to HD and HDS groups, the HDJ group had lower body and epididymal fat tissue weight gain and showed hypotrophy and hypoplasia. The RAS-related mRNA levels in the adipose tissue, including *Renin* and *Ace* were downregulated in the HDJ group compared to HD and HDS groups. Additionally, HDJ groups had significant improvements in systolic blood pressure, serum RAS-associated parameters (e.g., angiotensin II and aldosterone), renal mRNA levels related to RAS (e.g., angiotensin II receptor type 1 and 2), and aldosterone-associated mRNA expressions (e.g., mineralocorticoid receptor) in the kidney of HD-induced obese rats. Most importantly, HDS and HDJ groups showed distinct outcomes regarding adipogenesis and electrolytes metabolism, even though both diets contain a high level of salt. HDS group showed a higher epididymal fat tissue weight, mass, and adipocyte size than HDJ group. In addition, compared with HDJ group, HDS group significantly decreased the release of Na^+^ and K^+^ throughout the urine and feces. The present study addresses that *doenjang* has anti-obesity effects and anti-hypertensive effects by activating RAS in the adipose tissue and kidney, respectively. Additionally, this study also demonstrates that salt in *doenjang* and the additive salt differently influences adipogenesis and electrolytes metabolism, supporting *doenjang* has health advantageous effects regardless of its high salt contents.

## 1. Introduction

The imbalance between energy intake and expenditure results in obesity, which is one of the huge burdens for global health [1]; and the prevalence of obesity and obesity-related diseases have been increasing continuously [2]. For example, obesity is one of the major risk factors for hypertension [3]. A significant weight gain is strongly associated with increased blood pressure. Approximately 70% of hypertensive adults have excessive adipose tissues compared to normal individuals and obesity causes hypertension treatment resistance [2, 4].

The renin-angiotensin system (RAS) plays a pivotal role in the control of blood pressure also fluid and electrolyte balance through blood vessel constriction and reabsorption of Na^+^ and water [5]. Angiotensinogen (Agt) is the precursor of the bioactive angiotensin peptides; and angiotensin I (Ang I) and angiotensin II (Ang II) are produced from the subsequent cleavages of angiotensinogen by two key enzymes, renin and angiotensin-converting enzymes (Ace), respectively [6]. In addition to the roles in blood pressure, earlier studies have reported the significant involvement of RAS in obesity and insulin resistance because of its role in adipogenesis [7]. The level of *Agt* is positively associated with adipose tissue mass; and the upregulations of renin, Ace, and angiotensin II receptor type 1 (Agtr1) mRNA expression in the adipose tissue are observed in obesity [8, 9]. Indeed, activated RAS is commonly observed in metabolic syndrome patients, including obese subjects [10].

**Fig 1:**
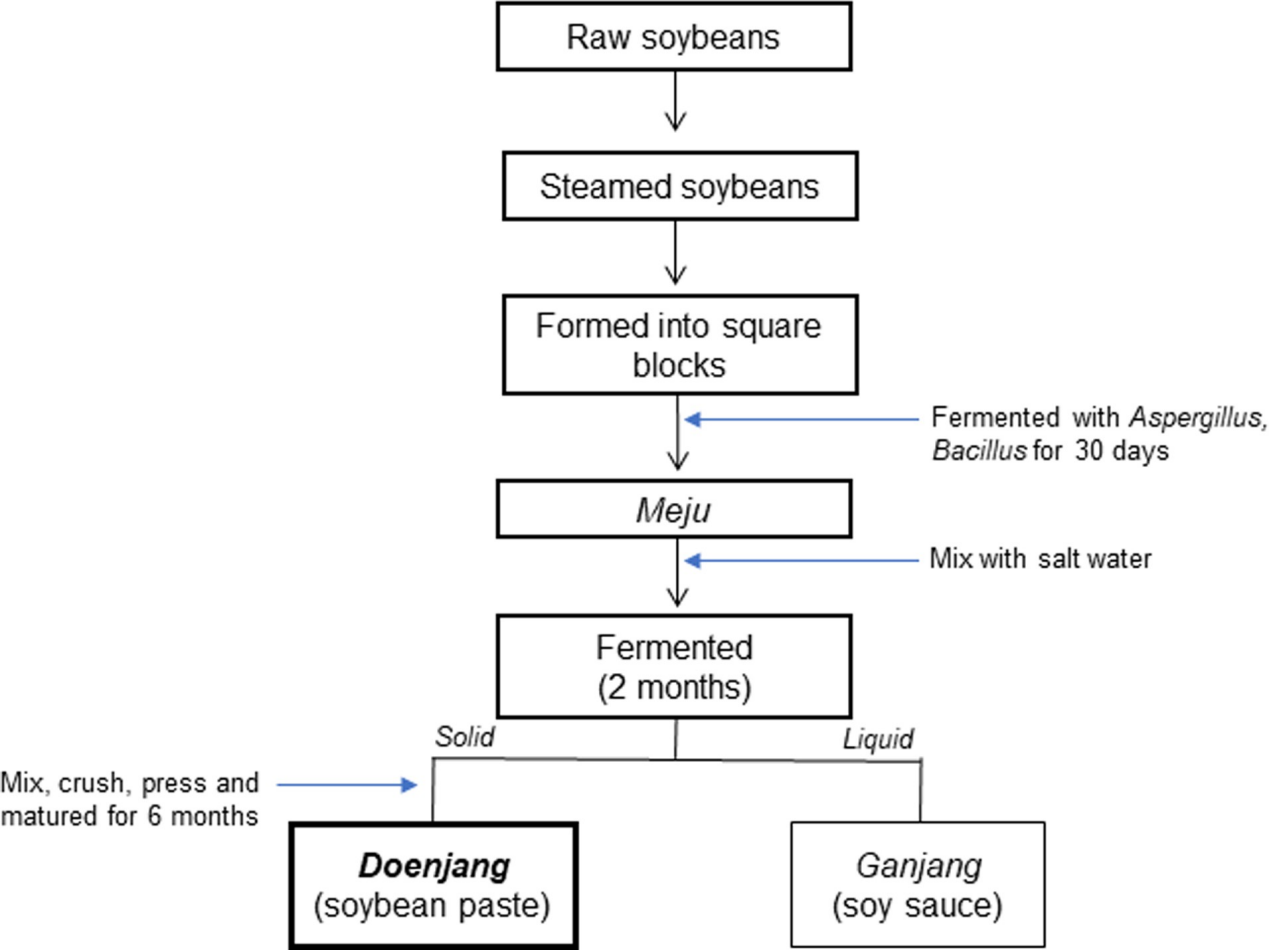
A diagram of manufacturing process of *Doenjang*. A recent study found that *doenjang* ameliorates high-salt diet-induced hypertension [16]. According to this study, the *doenjang*-fed group showed a significant reduction in blood pressure and a lower expression of sodium transferase-related genes compared to the group containing the same amount of salt in *doenjang* [16]. Both high salt diet and high fat diet have been considered as the major dietary factor for the hypertension; and interestingly, several publications presented that high-salt diet and high-fat diet have distinct impacts on the blood pressure, its related mechanisms, and the risk of hypertension [17, 18]. However, anti-hypertensive effects of *doenjang* in high-fat diet-induced hypertension are unexplored.

Korean traditional soybean paste, *doenjang* is one of the representative fermented products in South Korea. *Doenjang* is made with brine and fermented *Meju*, then mixed with salt (Fig 1). During the fermentation, the enzymes secreted by *Bacillus* break down the soybean protein and produce various amino acids and/or peptides, providing its own savory taste. Due to the fermentation and its components (e.g., soybean), *doenjang* has multiple health-beneficial effects, including anti-obesity effects [11–15]. According to a clinical study, the consumption of *doenjang* led to the anti-obesity and antioxidant effects, but also improved the overall health-related index in overweight individuals [12]. However, the exact underlying mechanisms of the anti-obesity effects of *doenjang* are not fully explored.

Although many previous studies reported the *doenjang*’s advantageous effects on the health, it is still controversial due to the high salt content in *doenjang*. This is because an excessive sodium intake increases the risk of hypertension via hypervolemia, promotes heart and kidney disease, and contributes to the development of gastric cancer [19, 20]. However, the addition of salt is essential in the manufacturing process of *doenjang*, as it inhibits harmful microbial growth, improves the survival of beneficial microbe, prolongs preservation, suppresses undesirable fermentation, and provides its unique tastes [14]. Therefore, it is critical to study the potential effects of high salt content in *doenjang* when the health beneficial effects of *doenjang* are investigated.

Therefore, the aims of this study were 1) to investigate whether *doenjang* influences RAS in adipose tissues to exert anti-obesity effects in HD-induced obesity, 2) to investigate the anti-hypertensive effects of *doenjang* in HD-induced obesity and its underlying mechanisms, and 3) to prove the high content of salt in *doenjang* does not have negative effects.

## 2. Materials and methods

### 2.1 Preparation of doenjang

*Doenjang* was produced by the Sunchang Sauce Corporation of South Korea (Sunchang-gun, Jeollabuk-do, Korea). This fermented food is prepared using soybean after maturing it for 6 months, by traditional Korean fermentation process (Fig 1). After steamed soybeans, it was made into blocks, and fermented with *Aspergillus oryzae* and *Bacillus subtilis* for one month. After this, it was mixed with brine (saltwater, 26%, w/v) in a 1:3 ratio and further fermented for 2 more months. Once matured, *doenjang* was dried using a freeze-dryer (FD12008, Ilshin Biobase, Gyeonggi-do, Korea), and its salinity was adjusted with NaCl (Samchun, Pyeongtaek-si, Gyeonggi-do, Korea) to 8%, using Mohr’s method [21].

For the cell treatment, 1 g of each freeze-dried *doenjang* was mixed with 10 mL of solvent (80% ethanol) at room temperature for 24 h on a shaker. The supernatants were collected and filtered through ADVANTEC No. 2 filter paper, and 1 mL of each filtrate was freeze-dried in a speed vacuum concentrator (FD12008, Ilshin Biobase, Gyeonggi-do, Korea).

### 2.2 Animals and treatments

Three-week-old male Sprague-Dawley rats were purchased from Central Lab. Animal, Inc. (Seoul, Korea), and acclimated at 12 h light and 12 h dark cycles at 25 ± 2°C and humidity 50% ± 5% conditions. After 7 days adaptation, they were randomly (no diffrence in body weight and initial SBP) divided into 4 groups (n = 6); normal diet control (ND; AIN76A, Research Diets, Inc. New Brunswick, NJ, USA), high-fat diet control (HD; 60% fat by weight, D12492, Research Diets, New Brunswick, NJ, USA), high-fat diet with 8% table salt (HDS), and a high-fat diet with *doenjang* containing 8% table salt (HDJ). All rats were fed with their corresponding experimental diets (Table 1) for 13 weeks, with water supplied. In addition, the nutritional composition of *doenjang* is shown in S1 Fig.

**Table 1:** The composition of experimental diets.

Ingredient	ND	HD	HDS	HDJ
Casein	20	25.85	23.96	22.93
L-Cystine	0.3	0.39	0.36	0.34
Corn Starch	15	0	0	0
Maltodextrin		16.15	14.97	14.33
Sucrose	50	8.89	8.24	7.89
Cellulose	5	6.46	5.99	5.73
Corn/Soybean Oil	5	3.23	2.99	2.87
Lard		31.66	29.35	28.08
Mineral Mix	3.5	1.29	1.20	1.15
Dicalcium Phosphate		1.68	1.56	1.49
Calcium Carbonate		0.71	0.66	0.63
Potassium Citrate		2.13	1.98	1.89
Vitamin Mix	1	1.29	1.20	1.15
Choline Bitartrate	0.2	0.26	0.24	0.23
NaCl (Table salt)			7.3	6.3
Dried *doenjang*				5
total	100	100	100	100
kcal/g	3.9	5.24	4.86	4.82

ND; AIN76A, Research Diets, Inc. New Brunswick, NJ, USA), (HD; 60% kcal% fat, D12492, Research Diets, New Brunswick, NJ, USA), (HDS; HD + 8% table salt), (HDJ; HD + *Doenjang* containing 8% salt)

Body weights were noted once a week and food intake were measured each day. Systolic blood pressure (SBP) was recorded weekly by the indirect tail-cuff method (BP-2000, Visitech Systems, Inc., Apex, NC, USA) 30 min after placing them at 37°C. The mean SBP was recorded after 7 measurements. After 10 weeks on each experimental diet, the rats were transferred to the metabolic cages (Med Associates Inc., VT, USA) for 3 days. Rats were kept under constant conditions and fed their respective diets. Urine and feces were collected daily from the metabolic cages into bottles. Additionally, food and water intake, and fecal and urine output were measured and collected for analyses. Mean value of each criterion was recorded for each rat. All animal procedures were approved by the Animal and Use Committee of Jeonbuk National University (JBNU 2018–052).

### 2.3 Tissue collection

At the end of 13 weeks, rats were fasted for 12 h before anesthetizing with 2 mg/kg BW alfaxalone (Alfaxan; Jurox, Australia) and 0.5 mL/kg BW xylazine (Rompun; Bayer, Seoul, Korea) through intramuscular injection to collect blood and tissues. Liver, epididymal adipose tissue, and one of the kidneys were rinsed with saline, weighed, and immediately frozen in liquid nitrogen and stored at -80°C for further analyses. The other kidney was fixed in 10% formaldehyde and embedded in paraffin. Blood was drawn by orbital vein puncture and centrifuged at 3,000 rpm for 15 min at 4°C to collect serum.

### 2.4 Biochemical analysis

A blood glucose test was conducted 10 weeks after rats were fed experimental diets. Blood samples were collected from the lateral tail vein after 12 hours of fasting. Blood glucose levels were measured by Accu Check Active Blood Glucose Meter Kit (Roche, Indianapolis, Indiana).

The serum levels of renin, angiotensin II and aldosterone were measured using assay kits (Rat Renin ELISA Kit, MyBioSource, San Diego, CA, USA; Angiotensin II ELISA Kit, and Aldosterone ELISA Kit, Enzo Life Sciences, Inc., Farmingdale, NY, USA), following the manufacturer’s protocols.

### 2.5 Histology of fat cryosections

Frozen epididymal adipose tissues were cryopreserved in OCT (Scigen Scientific Gardena, CA, USA), and frozen in liquid nitrogen. Sections of 10 μm thickness were cut with cryomicrotome (Shandon Cryotome FE, Thermo Scientific, MA, USA), transferred onto glass slide (Marienfeld, Germany) at -30°C. Sections were stained with hematoxylin and eosin (H&E) and mounted in glycerol gelatin. Cells were observed using an Axiophot Zeiss Z1 microscope (Carl Zeiss, Gottingen, Germany) at X200 magnification, and adipocytes were counted. Difference in cell size in each group were noted.

### 2.6 RNA extraction and real-time PCR

Total RNA was isolated after homogenizing the tissues in TRIzol reagent (Invitrogen, Grand Island, NY, USA), and the concentration of total RNA was equalized using quantifying on Biodrop Duo (Biochrom, Holliston, MA, USA). cDNA was synthesized using PrimeScript™ RT Master Mix (Takara Bio Inc., Shiga, Japan) according to the manufacturer’s instructions. RNA expression was measured by quantitative real-time polymerase chain reaction (qPCR) using the SYBR Green real-time PCR master mix (TOYOBO, Osaka, Japan), on a 7500 Real-Time PCR system (Applied Biosystems, Foster City, CA, USA), and quantitative analysis of PCR data were calculated through 2^−ΔΔCt^ method, using beta-actin as an internal control. Primers used for the qPCR are listed in Table 2.

**Table 2:** Sequences of primers used for PCR.

Gene	Forward	Reverse
*Pparγ*	ACCACTCGCATTCCTTTGAC	CCACAGACTCGGCACTCAAT
*Leptin*	TGACACCAAAACCCTCATCA	TCATTGGCTATCTGCAGCAC
*Adiponectin*	GCACTGGCAAGTTCTACTGCAA	GTAGGTGAAGAGAACGGCCTTGT
*Agt*	GATGCGCACAAGGTCCTG	CAGGGTGCTGTCCACACTGGCTCGC
*renin*	TTCTCTCCCAGAGGGTGCTA	CCCTCCTCACACAACAAGGT
*Ace*	GAGCCATCCTTCCCTTTTTC	GGCTGCAGCTCCTGGTATAG
*Agtr1*	ACTCTTTCCTACCGCCCTTC	TTAGCCCAAATGGTCCTCTG
*Agtr2*	GAAGGACAACTTCAGTTTTGC	CAAGGGGAACTACATAAGATGC
*Star*	GACCAGCCCATGGACAGACTC	AGGTCAATAGTGAGCAGCCA
*Hsd3b1*	ATGCCCAGTACCTGAGGAGA	TTGAGGGCCGCAAGTATCA
*Cyp11a1*	AGAAGCTGGGCAACATGGAGTCAG	TCACATCCCAGGCAGCTGCATGGT
*Cyp21*	CATCGTGCAACTAAGGCTAG	TGGAAGGGAGGAATTAAGAG
*MR*	GCTTTGATGGTAGCTGCG	TGAGCACCAATCCGGTAG
*Renalase*	TGACCTTGTCATCCTCACCA	AACTCCAAATGGGACAGTGG
*ß-actin*	AGCCTTCCTTCTTGGGTATGG	CACTTGCGGTGCACGGTATGG

*Pparγ*, Peroxisome proliferator-activated receptor-gamma; *Agt*, Angiotensinogen; *Ace*, Angiotensin-converting enzyme; *Agtr1*, Angiotensin II receptor type 1; *Agtr2*, Angiotensin II receptor type 2; *Star*, Steroidogenic acute regulatory protein; *Hsd3b1*, 3β-Hydroxysteroid dehydrogenase type 1; *Cyp11a1*, Cholesterol side-chain cleavage enzyme; *Cyp21*, 21-Hydroxylase; *MR*, Mineralocorticoid receptor.

### 2.7 Urine and feces analyses

Urine and feces samples, collected from metabolic cages, were analyzed for their Na^+^ and K^+^ contents, by inductively coupled plasma-mass spectroscopy (ICP-MS; 7500A, Agilent Technologies, Germantown, MD, USA), the center for University-Wide Research Facilities (CURF) at Jeonbuk National University.

### 2.8 Analyses of serum ion levels

The Na^+^ and K^+^ concentrations in the serum were determined using Fuji Dri-Chem Slide Na-K-Cl (FUJIFILM, Tokyo, Japan) with FDC 3500i chemistry analyzer (Fuji Dri-Chem Analyzer, Tokyo, Japan).

### 2.9. Cell culture and treatments

The 3T3-L1 preadipocyte cell line (CL‐173, ATCC, VA, USA) was maintained in DMEM (Hyclone, USA) containing 10% bovine serum (Gibco, NY, USA) and 100 U/mL 1% penicillin‐streptomycin (Hyclone, USA) at 37°C under 5% CO_2_ in a humidified incubator. RNA was extracted from 3T3-L1 cells at different times after differentiation.

To observe the effect of RAS blockers, the 3T3-L1 cells were seeded in 6-well plates and upon reaching 100% confluence (day 0), they were continued in culture for 48 h. Then the growth medium was replaced with differentiation medium, containing DMEM, 10% fetal bovine serum (FBS), 0.5 μM isobutylmethylxanthine (IBMX), 1 μM dexamethasone (DEXA), and 10 μg/mL insulin (Sigma‐Aldrich Co., St. Louis, MO, USA) with or without Losartan (10^−4^ M), Captopril (10^−4^ M), or *doenjang* (0.4% salinity). All treatments chemicals were dissolved in 30% EtOH to match the stock concentrations. The media, with or without the treatment chemicals, were changed every day, and the cells were harvested on the day 4. Total RNA was extracted using TRIzol reagent, according to the manufacturer’s instructions (Invitrogen) and quantified by quantitative real-time PCR with gene-specific primers (Table 2).

### 2.10 Statistical analyses

The data are expressed as mean ± SEM. By utilizing GraphPad Prism 8.0 (GraphPad Software, San Diego, CA, USA) or SPSS 17.0 (SPSS, Inc., Chicago, IL, USA), ANOVA and Tukey’s *post hoc* test, or unpaired t-test were conducted to detect significant differences between groups. The curve graph of body weight and systolic blood pressure were analyzed by two-way ANOVA with Tukey’s multiple comparisons. A *p*-value of less than 0.05 was considered significant.

## 3. Results

### 3.1 Doenjang improves body and fat tissue weight gains, and blood glucose levels in HD-induced obese rats

**Fig 2:**
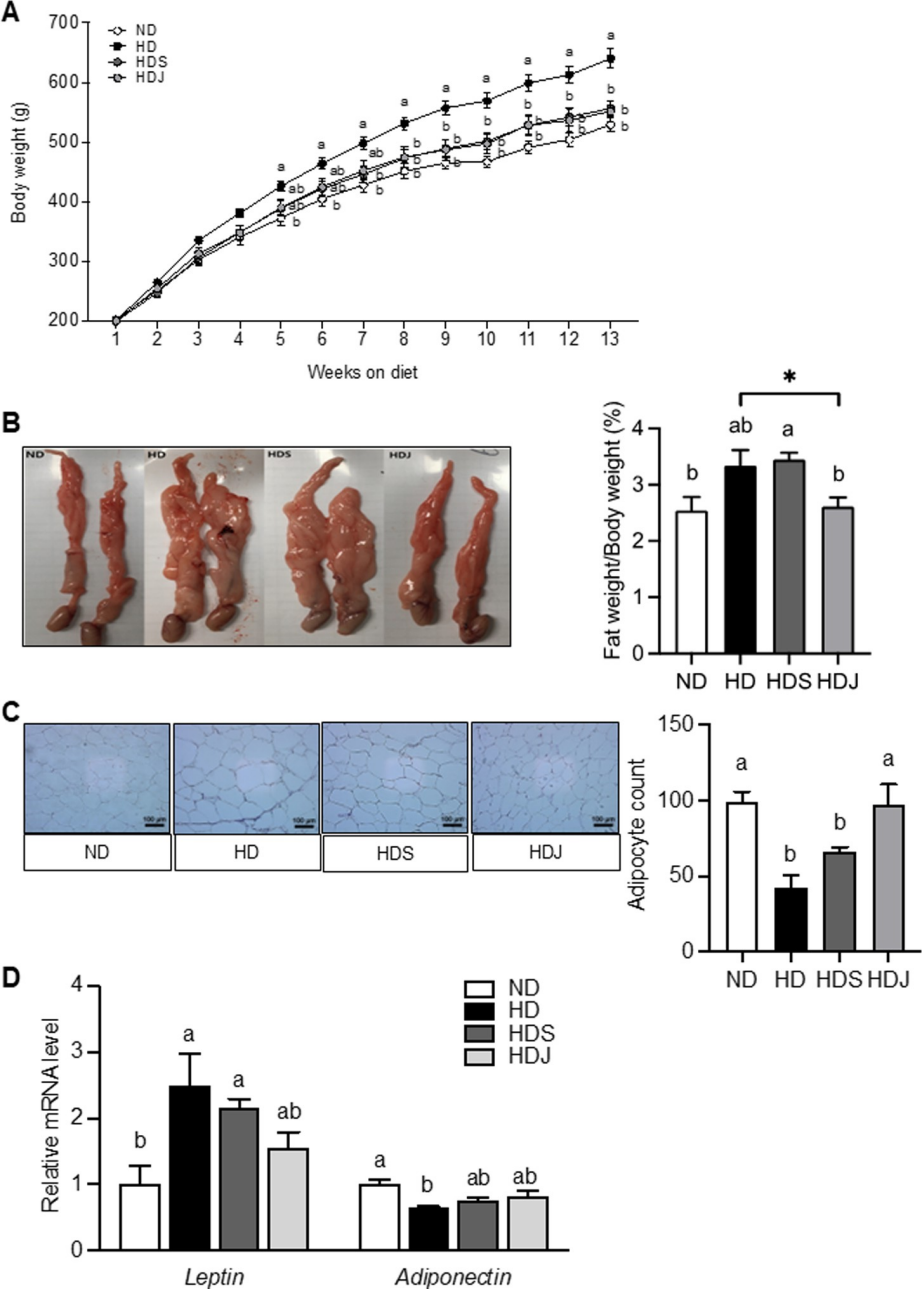
*Doenjang* reduces body and fat tissue weight gain in HD-induced obese rats. **(A)** A growth curve of body weight of each group is shown. The body weight was recorded weekly. **(B)** The pictures of representative epididymal fat tissues and a ratio of epididymal fat/body weight (%) are shown. **(C)** The histology sections of epididymal fat tissue and a ratio of the number of adipocytes to area are presented **(D)** The mRNA levels of leptin and adiponectin in epididymal fat tissue. Values are the mean ± SEM (n = 6) with different letters significantly different (p < 0.05) by ANOVA with Tukey’s post hoc test (a > b). Values with Asterisk present significant differences between the groups by unpaired t-test (*p < 0.05). ND; normal diet, HD; high-fat diet, HDS; high-fat diet with salt, and HDJ; high-fat diet with *doenjang*.

The initial body weight did not differ among different groups (Fig 2A and Table 3). The ND group had significantly lower body weight than HD group. Compared to HD group, HDJ group started to show a significant reduction of body weight from 8 weeks of the experimental period. On the final day of the experiment period, the HD group had significantly higher body weight compared to the ND group. Both HDS and HDJ groups showed a slightly elevated tendency of body weight compared to the ND group, but those elevations were markedly lower compared to the HD group (Fig 2A and Table 3). The diet intake of the ND group was significantly higher than all HD-fed groups (Table 3). Compared to the ND group, the blood glucose levels were significantly elevated in both HD and HDS groups, whereas the HDJ group had markedly attenuated blood glucose levels (Table 3).

**Table 3:** Body weight, diet intake, and blood glucose in rat.

	Initial Body Weight (g)	Final Body Weight (g)	Diet Intake (g/day)	Blood Glucose Level (mg/dL)
**ND**	202.95 ± 5.47^ns^	509.03 ± 10.88^c^	19.99 ± 0.39^a^	89.83 ± 2.36^b^
**HD**	201.82 ± 3.51^ns^	633.27 ± 17.20^a^	17.73 ± 0.46^b^	93.8 ± 4.19^a^
**HDS**	195.43 ± 8.61^ns^	558.65 ± 9.58^b^	17.02 ± 0.46^b^	90.33 ± 1.89^a^
**HDJ**	200.9 ± 4.73^ns^	548.48 ± 6.41^bc^	17.37 ± 0.42^b^	80.13 ± 2.86^c^

Values are given as mean ± SEM (n = 6). Values with different superscripts in the same column are significantly different (p < 0.05) by Duncan’s multiple range test (a > b; ns, not significant). ND; normal diet, HD; high-fat diet, HDS; high-fat diet with salt, and HDJ; high-fat diet with *doenjang*.

The relative epididymal fat weight ratio of the HD and HDS groups was significantly higher than the ND groups, while the HDJ group had markedly lower epididymal fat mass and the levels of the relative epididymal fat weight ratio compared to both HD and HDS groups (Fig 2B). Moreover, the HDJ group showed a significantly reduced adipocyte size and an increased ratio of the number of adipocytes to the area compared to HD and HDS groups (Fig 2C). Consistent with these results, the HDJ group showed a downregulated tendency of leptin mRNA expression and an upregulated tendency of adiponectin mRNA level in epididymal adipose tissues compared to HD and HDS groups without statistical significance (Fig 2D). These observations suggest that the consumption of *doenjang* ameliorates HD-induced obesity by inhibiting body weight gain and adipogenesis, and by improving obesity-related gene expression in adipose tissue.

### 3.2 Doenjang changes RAS-related gene levels in the liver and adipose tissue in HD-induced obese rats

The elevations of RAS-related gene expression and RAS activation in the adipose tissues are associated with obesity; for example, Agtr1 and Agtr2 regulate adipogenesis [9, 22]. Previously, *doenjang* significantly reduces the relative adipose tissue weight ratio, adipocyte size, and increased ratio of the number of adipocytes to the area in HD-induced obese rats (Fig 2B and 2C). Therefore, hepatic *Agt* and RAS-associated gene expressions in the adipose tissues were analyzed to scrutinize whether *doenjang* influences adipogenesis via RAS.

**Fig 3:**
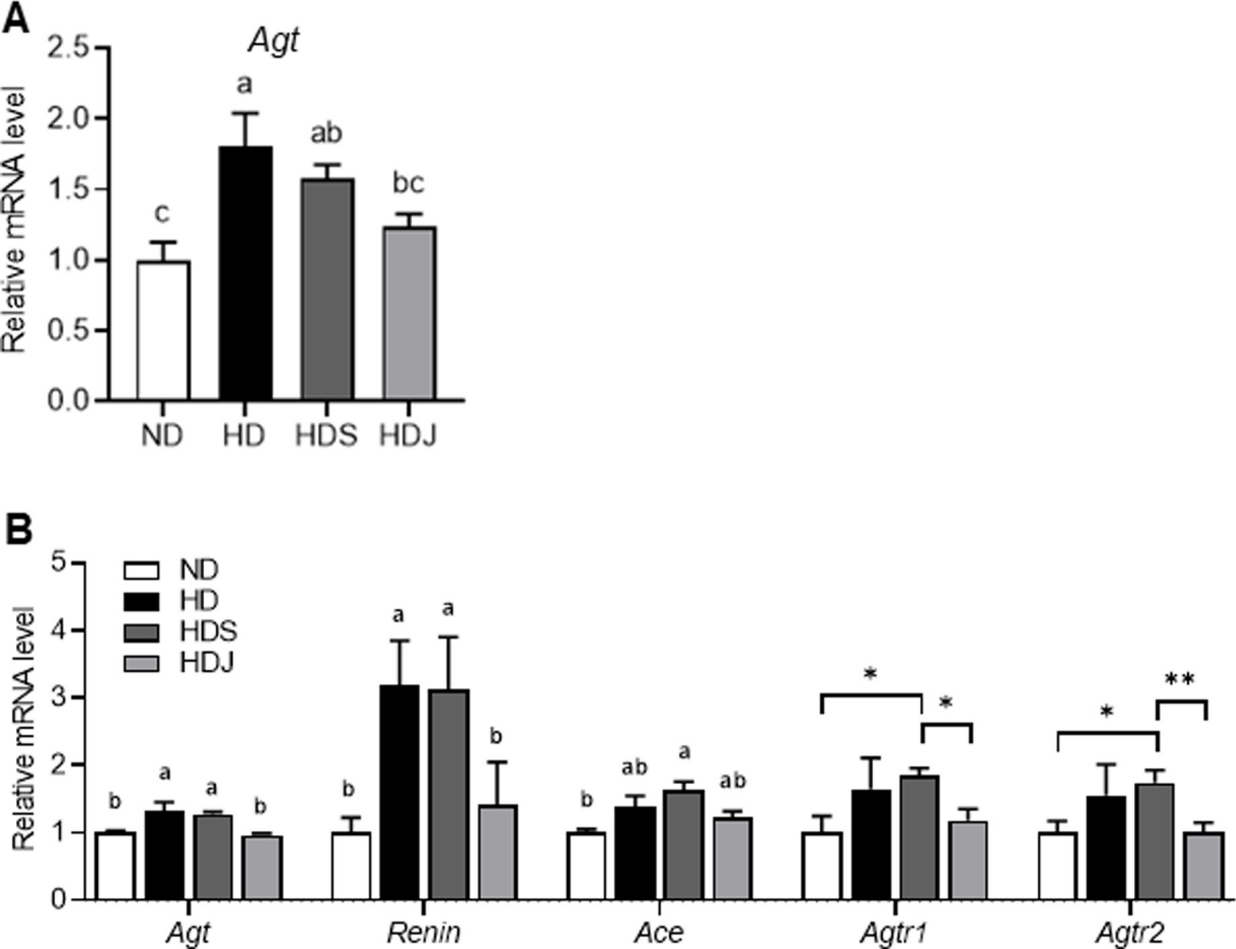
*Doenjang* alters the mRNA expression related to RAS in the liver and white adipose tissue in HD-induced obese rats. **(A)** mRNA level of hepatic *Agt*. **(B)** mRNA levels of RAS-related genes in white adipose tissue. Values are given as mean ± SEM (n = 6). Values with different superscripts are significantly different (p < 0.05) by ANOVA with Tukey’s post hoc test (a > b). Values with Asterisk present significant differences between the groups by unpaired t-test (*p < 0.05 and **p<0.01). ND; normal diet, HD; high-fat diet, HDS; high-fat diet with salt, and HDJ; high-fat diet with *doenjang*. *Agt*; Angiotensinogen, *Ace*; Angiotensin-converting enzyme, *Agtr1*; Angiotensin II receptor type 1, and *Agtr2*; Angiotensin II receptor type 2.

Compared to the ND group, the hepatic *Agt* expression was significantly upregulated in HD and HDS groups; while the HDJ group had significantly decreased hepatic *Agt* level compared to the HD group (Fig 3A). In addition, all RAS-associated gene expressions, including *Agt*, *Renin*, *Ace*, *Agtr1*, and *Agtr2* in the adipose tissue were higher in both HD and HDS groups compared to the ND group with and/or without statistical significance (Fig 3B). Consistent with the results in Fig 2B and 2C, the levels of the above genes in the HDJ group were lower than HD and HDS groups with and/or without statistical significance (Fig 3B).

**Fig 4:**
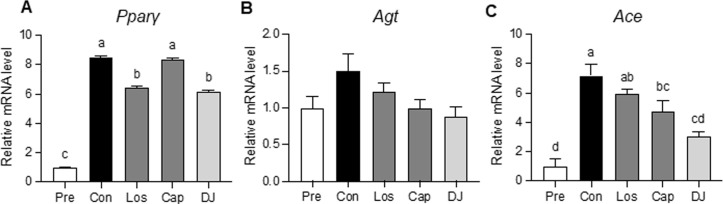
The effects of d*oenjang* in differentiated 3T3L1 cells. Differentiated 3T3L1 cells were treated with losartan (Los, 10^−4^ M), captopril (Cap, 10^−4^ M), and Doenjang (DJ, 0.4% salinity) for 4 days. **(A)** The mRNA levels of *Pparγ* **(B)** The mRNA levels of *Agt*. **(C)** The mRNA levels of *Ace*. Values with different superscripts are significantly different (p < 0.05) by ANOVA with Tukey’s post hoc test (a > b). Pre; preadipocytes, Con; differentiated adipocyte, *Pparγ*; peroxisome proliferator-activated receptor gamma, *Agt*, Angiotensinogen, *Ace*; Angiotensin-converting enzyme.

To further investigate the direct effects of *doenjang* on adipogenesis and RAS-related gene expressions, the differentiated 3T3L1 cells were treated with Losartan (Los, Agtr inhibitor), Captopril (Cap, Ace inhibitor), and *doenjang* extraction (0.4% salinity). Treatments of *doenjang* and Los significantly reduced the key adipogenic transcription factor, *Pparγ* in the differentiated 3T3L1 cells (Fig 4A). Moreover, *doenjang* and Cap treatments decreased *Agt* without statistical significance and significantly lowered *Ace* expressions in the differentiated 3T3L1 cells (Fig 4B and 4C). These findings indicate that *doenjang* downregulates the expressions of RAS-related gene and adipogenesis-associated gene in the adipose tissue, resulting in the reduction of adipogenesis and the improvements of obesity, subsequently.

### 3.3 Doenjang improves systolic blood pressure and serum RAS-related parameters in HD-induced obese rats

**Fig 5:**
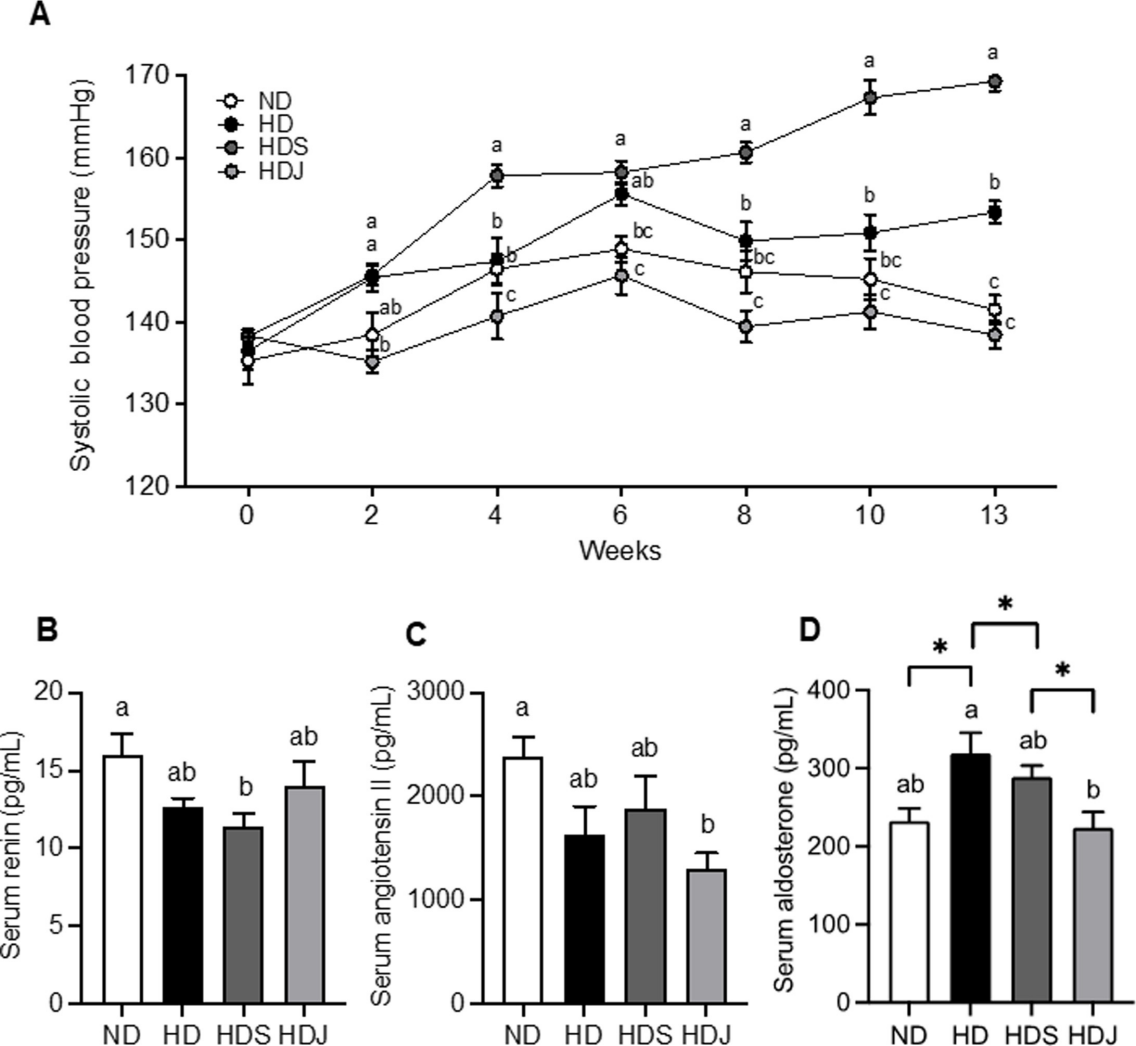
*Doenjang* ameliorates systolic blood pressure and serum RAS-associated indicators in HD-induced obese rats. **(A)** A periodical changes of systolic blood pressure during the experimental period are shown. Values are given as mean ± SEM (n = 4) **(B)** The level of serum renin. **(C)** Serum angiotensin II level. **(D)** Serum aldosterone level. Values are given as mean ± SEM (n = 6). Values with different superscripts are significantly different (p < 0.05) by ANOVA with Tukey’s post hoc test (a > b). Values with Asterisk present significant differences between the groups by unpaired t-test (*p < 0.05). ND; normal diet, HD; high-fat diet, HDS; high-fat diet with salt, and HDJ; high-fat diet with *doenjang*.

Next, the parameters involved in hypertension were studied to investigate the anti-hypertensive effects of *doenjang* in HD-induced obesity. The initial SBP of all groups was 125 ± 10 mmHg, which was continuously and significantly increased over the experimental period, especially in both HD and HDS groups (Fig 5A). After 2 weeks, HD the blood pressure of HD and HDS groups became significantly higher than HDJ group. At 8 week, HDS group had the highest blood pressure among all groups, while HDJ group showed the lowest level of blood pressure despite of the fact that HDJ diet contains the same amount of salt as the HDS diet. ND group had the highest serum renin level among the groups (Fig 5B). Compared to the ND group, both HD and HDS groups had lower serum renin levels with and/or without statistical significance, whereas the HDJ group had a significantly higher serum renin level than the HDS group (Fig 5B). Compared to the ND group, both HD and HDS groups showed a decreased tendency of serum angiotensin II levels, while the HDJ group had a significant reduction of serum angiotensin II levels (Fig 5C). The levels of serum aldosterone were significantly increased in both HD and HDS groups compared to the ND group, and the HDJ group had markedly reduced levels of serum aldosterone compared with HD and HDS groups (Fig 5D). These findings imply *doenjang* intake could improve hypertension in HD-induced obesity via the ameliorations of RAS-related serum indicators.

### 3.4 Doenjang alters RAS-related gene expressions in the kidney in HD-induced obese rats

**Fig 6:**
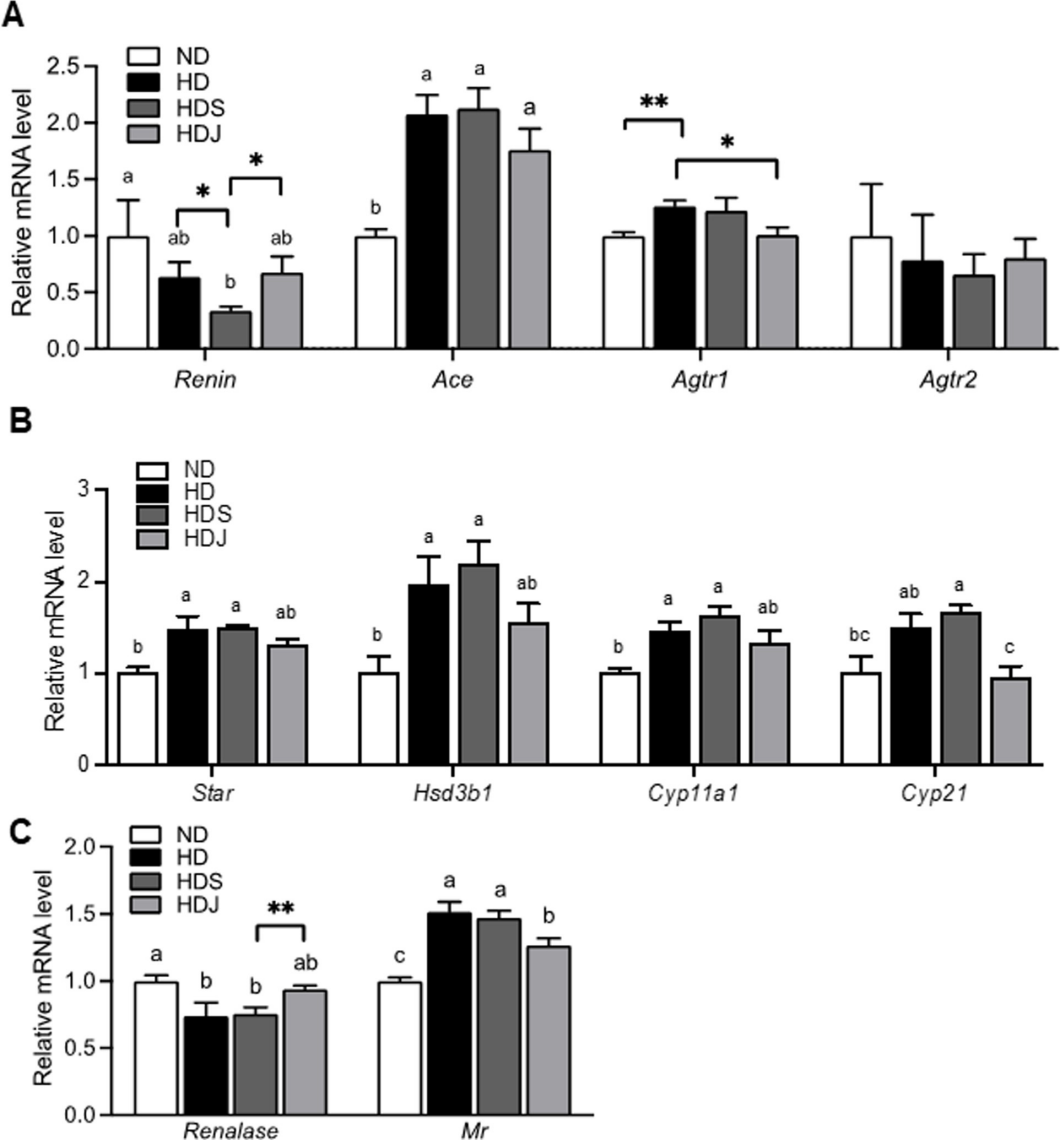
*Doenjang* changes renal mRNA levels related to RAS in HD-induced obese rats. **(A)** mRNA levels of RAS-related genes in the kidney. **(B)** Aldosterone releasing factors-related mRNA levels in the kidney. **(C)** The levels of Mr and Renalase mRNA in the kidney. Values are given as mean ± SEM (n = 6). Values with different superscripts are significantly different (p < 0.05) by ANOVA with Tukey’s post hoc test (a > b). Values with Asterisk present significant differences between the groups by unpaired t-test (*p < 0.05 and **p<0.01). ND; normal diet, HD; high-fat diet, HDS; high-fat diet with salt, and HDJ; high-fat diet with *doenjang*. *Ace*; Angiotensin-converting enzyme, *Agtr1*; Angiotensin II receptor type 1, *Agtr2*; Angiotensin II receptor type 2, *Star*; Steroidogenic Acute Regulatory Protein, *Hsd3b1*; 3β-hydroxysteroid dehydrogenase type 1, *Cyp11a1*; cholesterol side-chain cleavage enzyme, *Cyp21*; 21-hydroxylase, and *Mr*; Mineralocorticoid receptor.

As *doenjang* improves SBP and serum RAS-associated parameters (Fig 5), the expressions of RAS-involved genes in the kidney were measured. The Renin mRNA expression in the kidney, a feedback controller of Agt was decreased in both HD and HDS groups compared to ND groups without statistical significance (Fig 6A). Compared to the HD groups, the HDS group had a significant reduction of Renin mRNA level, while the HDJ group showed a significantly higher expression of Renin mRNA compared to the HDS groups (Fig 6A). Compared to the ND group, all HD groups showed a significantly elevated level of renal *Ace*, but the HDJ group had a slightly reduced *Ace* level compared to HD and HDS groups (Fig 6A). The expression of renal *Agtr1* was increased in both HD and HDS groups with and/or without statistical significance, but it was reduced in the HDJ group compared to the HD and HDS groups with and/or without statistical significance (Fig 6A). The level of *Agtr2* in the kidney did not significantly change among the groups; however, both HD and HDS groups had a lower tendency than ND group, while HDJ group had an elevated trend compared with HD and HDS groups (Fig 6A).

Additionally, aldosterone-associated genes in the kidney, including *Star*, *Hsd3b1*, *Cyp11a1*, and *Cyp21* were analyzed. Compared to the ND group, HD and HDS groups had significant upregulation of *Star*, *Hsd3b1*, *Cyp11a1*, *and Cyp21* levels (Fig 6B). HDJ group showed a slight downregulated tendency of *Star*, *Hsd3b1*, and *Cyp11a1* expressions compared to both HD and HDS groups without statistical significance; however, the level of *Cyp21* was significantly downregulated in the HDJ group compared to HD and HDS groups (Fig 6B).

A deficiency of renalase is linked with the elevated blood pressure; and mineralocorticoid receptor (MR) plays an essential role in aldosterone functions [23, 24]. Both HD and HDS groups had significantly downregulated renalase mRNA levels compared to the ND group, whereas the HDJ group showed markedly upregulated Renalase mRNA expression compared to HDS groups (Fig 6C). The mRNA expression of Mr was significantly elevated in both HD and HDS groups, while the HDJ group had significantly lower Mr mRNA expression compared to HD and HDS groups (Fig 6C). These results address that *doenjang* changes the renal gene expressions related to RAS and aldosterone in HD-induced obese rats, which explains how *doenjang* has anti-hypertensive effects against hypertension coming from HD-induced obesity.

### 3.5 Doenjang modifies urine excretion, water intake, and electrolyte ratio in HD-induced obese rats

As *doenjang* changes serum RAS-associated parameters (Fig 5B–5D) and the renal mRNA expression related to RAS and aldosterone (Fig 6), the effects of *doenjang* on fluid and electrolyte balance, urine excretion, and water intake were analyzed. Additionally, Na^+^ and K^+^ levels in urine, feces, and serum were also measured.

**Fig 7:**
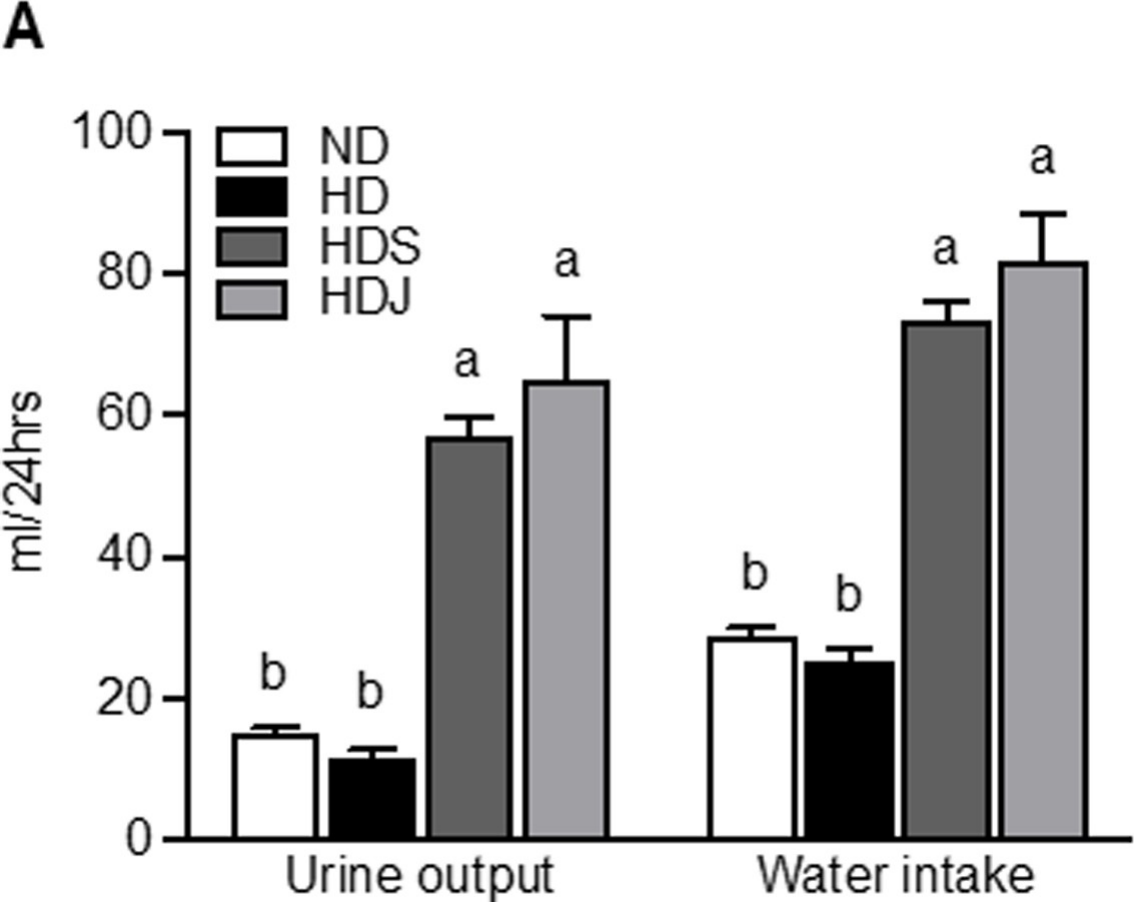
*Doenjang* increases urine output and water intake in HD-induced obese rats. The output of urine and water intake were measured in the metabolic cages for 24 hours. Values are given as mean ± SEM (n = 6). Values with different superscripts are significantly different (p < 0.05) by ANOVA with Tukey’s post hoc test (a > b). ND; normal diet, HD; high-fat diet, HDS; high-fat diet with salt, and HDJ; high-fat diet with *doenjang*.

As expected, urine output and water intake were significantly higher in both HDS and HDJ groups compared to ND and HD groups (Fig 7). HDS and HDJ groups had a significantly higher level of urinary Na^+^ compared to ND and HD groups; however, the urinary K^+^ level was significantly higher in the HDJ group than in the HDS group (Table 4). As a result, the HDS group had the highest urinary Na^+^/K^+^ ratio among all groups, whereas HDJ group had a markedly lower urinary Na^+^/K^+^ ratio compared to the HDS group. Additionally, the HDJ group had an elevated tendency of the fecal Na^+^ level compared to both HD and HDS groups, but there were no statistically significant differences among all groups (Table 4). The level of fecal K^+^ was markedly higher in the HDJ group compared with the HDS group. Consequently, the HDS group showed the highest fecal Na^+^/K^+^ ratio among the groups, while the HDJ group had a significantly reduced fecal Na^+^/K^+^ ratio compared to the HDS group (Table 4). All HD groups had an increased tendency of the serum Na^+^ level compared to ND group, but HDJ group showed a slight reduction of serum Na^+^ level compared to HD and HDS groups (Table 4). The serum K^+^ level of HD and HDS groups was significantly higher than ND groups, whereas HDJ group had a markedly decreased serum level of K^+^ compared to HD and HDS groups. These observations suggest that *doenjang* improves hypertension in HD-induced obese rats by increasing water excretion and elimination of Na^+^ and K^+^ through urine and feces.

**Table 4:** Na^+^ and K^+^ levels of urine, feces, and serum in rats.

	Urine (ppm)			Feces (ppm)			Serum (mEq/L)	
	Na^+^	K^+^	Na^+^/K^+^	Na^+^	K^+^	Na^+^/K^+^	Na^+^	K^+^
**ND**	1845.86 ± 134.35^d^	4929.48 ± 461.13^b^	0.42 ± 0.02^c^	504.08 ± 41.96^ns^	1098.18 ± 100.55^a^	0.48 ± 0.37^c^	134.7 ± 0.85^b^	4.34 ± 0.11^b^
**HD**	2431.53 ± 158.41^c^	12571.03 ± 469.02^a^	0.19 ± 0.01^d^	548.99 ± 98.73^ns^	728.30 ± 68.08^b^	0.43 ± 0.42^c^	138.3 ± 1.31^a^	5.43 ± 0.17^a^
**HDS**	9315.33 ± 32.78^a^	1870.31 ± 67.18^d^	4.46 ± 0.00^a^	568.70 ± 2.70^ns^	462.66 ± 39.11^c^	1.59 ± 0.60^a^	137.5 ± 0.43^ab^	5.62 ± 0.12^a^
**HDJ**	9426.76 ± 36.69^a^	2183.75 ± 64.67^c^	3.83 ± 0.00^b^	614.53 ± 2.47^ns^	828.92 ± 36.06^b^	0.84 ± 0.77^b^	136.8 ± 1.05^ab^	4.72 ± 0.21^b^

Values are given as mean ± SEM (n = 6). Values with different superscripts in the same column are significantly different (p < 0.05) by Duncan’s multiple range test (a > b; ns, not significant). ND; normal diet, HD; high-fat diet, HDS; high-fat diet with salt, and HDJ; high-fat diet with *doenjang*.

## 4. Discussion

*Doenjang*, a Korean traditional soybean paste has diverse health-beneficial effects [11–13], although it has been contentious due to the high salt content in *doenjang*. Moreover, most of the previous studies regarding the health-advantageous effects of *doenjang* (e.g., anti-obesity and anti-hypertensive effects) did not fully present its underlying mechanisms. The present study found that *doenjang* reduces adipogenesis in HD-induced obese rats via the downregulation of RAS-associated gene levels in adipose tissue. Additionally, *doenjang* elicits anti-hypertensive effects by ameliorating renal RAS in HD-induced obese rats. Most importantly, *doenjang* shows these beneficial effects nevertheless its high salt contents.

It has been reported that the dietary sodium in the HD diet lowers body weight gain due to the reduction of digestive efficiency coming from RAS inhibition by sodium [25]. Even though high sodium intake did not elevate body weight gain, it markedly increases fat accumulation [26]. Consistent with the earlier reports, the present study found that the supplementations of additive salt in the HD diet do not increase the body weight gain but, significantly elevate the relative epididymal fat weight ratio, epididymal fat mass, and adipocyte size. In contrast, *doenjang* significantly improved those indicators but also the mRNA levels of leptin and adiponectin in HD-induced obese rats, suggesting the anti-obesity effects of *doenjang* against HD-induced obesity. Many previous studies have been suggesting that soybean-based Korean fermented elicits its health beneficial outcomes due to its components, although the exact compound is not fully established. As a major component of *doenjang*, soybean contains multiple bioactive compounds, such as saponin, isoflavones, and peptides [27, 28]. Therefore, future studies should investigate the potential and/or exact compounds in *doenjang* that play role in its anti-obesity effects. Moreover, like many previous studies [29, 30], the current study did not have doenjang-only supplemented group to investigate the effects of doenjang itself. Thus, it will be also essential to set up doenjang-only supplemented group in the future studies to concrete understanding of its health beneficial effects.

Obesity activates RAS and increases RAS-related gene expressions in the adipose tissue, including *Agt*, *Renin*, *Ace*, and *Agtr1* [8, 9]. Ang II increases adipogenesis by activating *Agtr1* and *Agtr2*, resulting in lipolysis reduction and lipogenesis elevation, respectively [22]. This study reported that the supplementation of *doenjang* downregulates hepatic *Agt* and RAS-associated genes in the adipose tissue in HD-induced obese rats. Moreover, *doenjang* directly decreased the adipogenesis-related gene expression (e.g., *Pparγ*) and RAS-related gene levels (e.g., *Agt* and *Ace*) in differentiated 3T3L1 cells. In summary, these findings address that the hypotrophy and hypoplasia of adipocytes by *doenjang* came from the reductions of RAS-related gene expression in the adipose tissue. According to the earlier findings, Ang II activates phosphoinositide 3-kinase (PI3K)/Akt and mitogen-activated protein kinase (MAPK) signaling pathways in the adipose tissue to increase adipocyte proliferation and adipogenic differentiation, respectively [31]. Thus, future studies regarding the activation of RAS-adipogenesis-related signaling pathways by *doenjang* are essentially required to understand its underlying molecular and cellular mechanisms.

Excessive fat accumulation is strongly associated with blood pressure elevation and the risk of hypertension [32, 33]. For example, adipocyte-derived angiotensin activates Ang II, leading to the vasoconstriction and activation of aldosterone [34]. As aldosterone stimulates the reabsorption of Na^+^ and K^+^ release, activation of aldosterone contributes to the elevation of blood pressure [34, 35]. In the present study, as expected, the HD diet and the additive salt supplemented HD diet significantly increased SBP, while *doenjan*g did not elevate SBP in the HD-induced obese rats. Moreover, *doenjang* improved the levels of serum RAS-related indicators (e.g., Ang II and aldosterone), mRNA expressions involved in renal RAS (e.g., *Ace* and *Agtr1*) and the functions of aldosterone (e.g., *Cyp21* and *Mr*). A previous study reported that soybean bioactive peptide strongly improved kidney structure, antioxidant defense system, and inflammation in hypertensive rats [36]. Hence, the effects of *doenjang* consumption on overall kidney structure, renal antioxidant defense system, and renal inflammatory response are needed in the future to study another possible key mechanism of *doenjang*’s anti-hypertensive outcomes.

Although Korean consumes high salt from their traditional fermented foods, including *doenjang*, the prevalence of obesity and hypertension is relatively lower than in other countries [37]; and this phenomenon is defined as the “Korean Paradox” [38, 39]. Korean Paradox proposes the possibility that the metabolism and functions of the salt in *doenjang* and the additive salt may differ. The present study found that the additive salt induces hypertrophy and hyperplasia, while *doenjang* shows opposite outcomes in HD-induced obese rats, even though both diets contain the same amounts of salt. Due to the high salt contents, *doenjang* and the additive salt supplementation significantly increased water intake and urine output; however, *doenjang* markedly elevated the excretions of Na^+^ and K^+^ via urine and feces; therefore, the ratio of Na^+^/K^+^ was markedly lowered by *doenjang* than the additive salt supplementation. As a lower Na^+^/K^+^ ratio is associated with a lesser risk of hypertension [40], the findings in this study address that *doenjang* ameliorates hypertension in HD-induced obesity by altering electrolyte metabolism and its excretion. Moreover, these observations support the potential differences in salt metabolism that is contained in *doenjang* and the additive salt. Thus, in the future, more detailed underlying mechanisms regarding the metabolism of salt in *doenjang* should be investigated.

## Supporting information

S1 Fig The nutritional composition of *doenjang*. (TIF)

## Abbreviations

Ace: Angiotensin-converting enzymes
Agt: Angiotensinogen
Agtr1: Angiotensin II receptor type 1
Agtr2: Angiotensin II receptor type 2
Ang I: Angiotensin I
Ang II: Angiotensin II
Cyp11a1: cholesterol side-chain cleavage enzyme
Cyp21: 21-hydroxylase genes
Hsd3b1: 3β-hydroxysteroid dehydrogenase type 1
Mr: Mineralocorticoid receptor
Pparγ: Peroxisome proliferator-activated receptor gamma
RAS: Renin-angiotensin system
SBP: Systolic blood pressure
Star: Steroidogenic acute regulatory protein
