# Supplementary figures and images for: Korean fermented soybean paste (Doenjang) has anti-obesity and anti-hypertensive effects via the renin-angiotensin system (RAS) in high-fat diet-induced obese rats
Source: PLoS One. 2023 Oct 20;18(10):e0291762. doi: 10.1371/journal.pone.0291762 (PMC10588895; doi:10.1371/journal.pone.0291762)

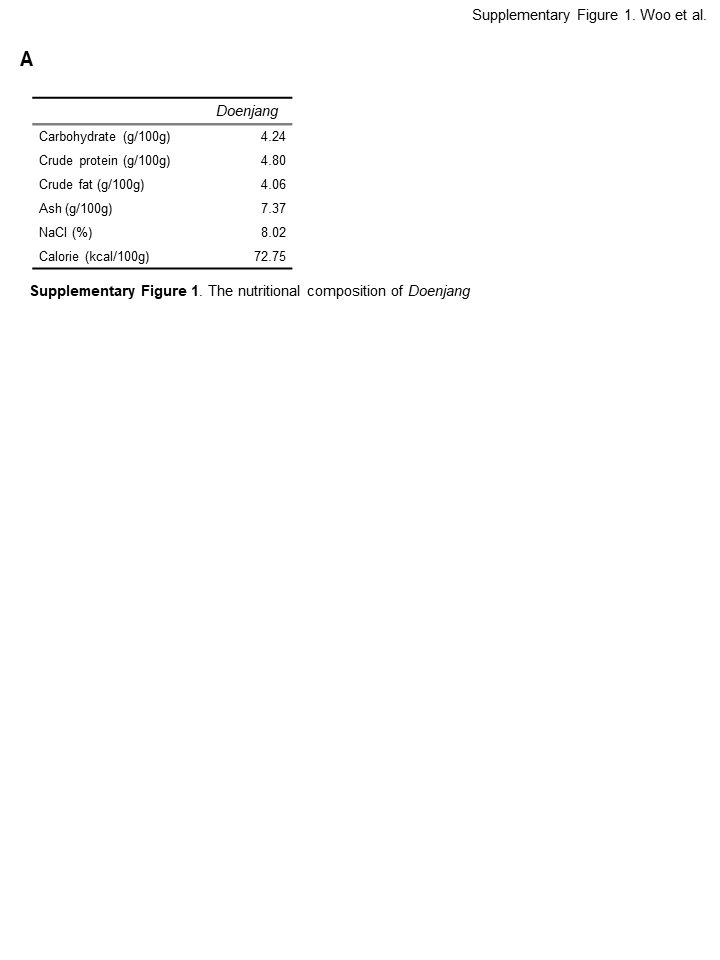

Supplement: S1 Fig — (TIF) [file pone.0291762.s001.tif]
